# Strong attenuation of SARS-CoV-2 Omicron BA.1 and increased replication of the BA.5 subvariant in human cardiomyocytes

**DOI:** 10.1038/s41392-022-01256-9

**Published:** 2022-12-25

**Authors:** Rayhane Nchioua, Federica Diofano, Sabrina Noettger, Pascal von Maltitz, Steffen Stenger, Fabian Zech, Jan Münch, Konstantin M. J. Sparrer, Steffen Just, Frank Kirchhoff

**Affiliations:** 1 Institute of Molecular Virology, Ulm University Medical Center, Ulm, Germany; 2 Molecular Cardiology, Department of Internal Medicine II, Ulm University, Ulm, Germany; 3 Institute of Medical Microbiology and Hygiene, Ulm University Medical Center, Ulm, Germany

**Keywords:** Infection, Infectious diseases

**Dear Editor**,

Since its first description in South Africa in November 2021, the SARS-CoV-2 Omicron variant rapidly outcompeted the previously dominating Delta variant. Omicron is the fifth variant of concern (VOC). It contains an unusually high number of mutations compared to previous VOCs, especially in the viral Spike protein, and shows high transmissibility and efficient escape of neutralizing antibodies. Due to these characteristics, it received this designation much faster than the four previous VOCs Alpha, Beta, Gamma, and Delta. However, the original BA.1 Omicron variant seems to be less pathogenic than early SARS-CoV-2 strains and other VOCs.^1^ While SARS-CoV-2 primarily infects the respiratory tract, Coronavirus disease 19 (COVID-19) is a multi-organ disease, and patients show infection and disorders in the gastrointestinal, cardiovascular, and neurological systems. Thus, the ability of the various SARS-CoV-2 variants to infect and propagate in different cell types and organs clearly plays a key role in viral pathogenicity. Especially, cardiomyocytes express high levels of the primary SARS-CoV-2 receptor ACE2 and are highly permissive for viral replication.^2^

Cardiac injury and cardiomyopathies are common complications of COVID-19. Clinical manifestations leading to severe or even fatal outcomes include myocarditis, heart failure, arrhythmia, and Takotsubo cardiomyopathy (TCM).^3^ The mechanism(s) underlying heart injury in COVID-19 are not entirely clear. Direct effects of SARS-CoV-2 on cardiomyocytes are supported by their high susceptibility to virus infection and detection of viral RNA and Spike protein in autopsy cardiac tissues of COVID-19 patients.^3^ In addition, it has been shown that SARS-CoV-2 infects and efficiently replicates in cardiomyocytes but not in cardiac macrophages, fibroblasts, or endothelial cells.^4,5^ The BA.1 Spike shows altered ACE2 affinity, reduced dependency on TMPRSS2 for proteolytic activation, changes in cell tropism and reduced fusogenicity compared to the original HU-1 strain and the Delta VOC.^6^ However, it is currently not known whether early SARS-CoV-2 strains, Delta and Omicron BA.1, differ in their replication fitness, cytopathicity and fusogenicity in human cardiomyocytes.

To determine their susceptibility to viral replication and cytopathic effects, beating iPSC-derived human cardiomyocytes were infected with three different multiplicities of infection (MOI) of the early NL-02-2020 strain of SARS-CoV-2, Delta (B.1.617.2), or Omicron (BA.1, B.1.1.529) VOCs. These cultures consist of >90% ventricular cardiomyocytes (Supplementary Fig. 1).^7^ In agreement with previous data,^2^ cardiomyocytes were highly susceptible to SARS-CoV-2 replication (Fig. 1a). Infection at lower MOI (0.01 or 0.1) was associated with moderately delayed viral replication kinetics. However, virus production generally achieved a similar maximum at about 5 days post-infection (Supplementary Fig. 2). On average, the NL-02-2020 and Delta strains reached significantly higher levels of viral RNA (Fig. 1a) and produced about 2–3 orders of magnitude more infectious virions (Fig. 1b) than BA.1. In agreement with increased fusogenicity, the Delta variant caused stronger cytopathic effects (CPE) than NL-02-2020, while BA.1 induced only modest and delayed CPE (Supplementary Fig. 3). Confocal microscopy analysis of infected cultures at 3 dpi revealed that those infected with NL-02-2020 and Delta lost their well-organized cardiac Troponin T-positive sarcomeric structure (Fig. 1c, Supplementary Fig. 4). In contrast, the sarcomeric structure was preserved in BA.1-infected cultures.

**Fig. 1:**
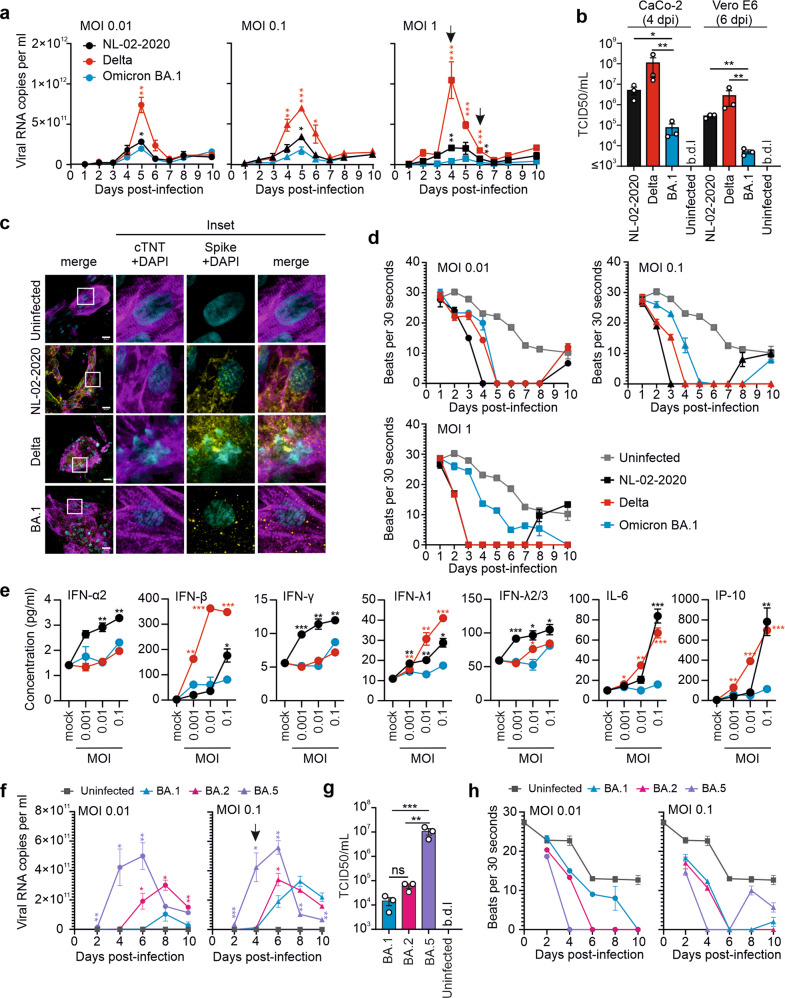
Replication of SARS-CoV-2 NL-02-2020, Delta and Omicron variants in human cardiomyocytes. **a** Replication kinetics of the SARS-CoV-2 NL-02-2020, Delta and Omicron BA.1 variants in human cardiomyocytes at the indicated days post-infection. **b** Infectious SARS-CoV-2 particles in the supernatants of cardiomyocytes infected at an MOI of 1 at 4 and 6 days post-infection (indicated by arrows in panel **a**). TCID50 was determined by infection of CaCo-2 (day 4 pi) and Vero E6 (day 6 pi) cells as described in the methods section. b.d.l, below detection limit. **c** Confocal images showing the effect of SARS-COV-2 variants infection on cardiomyocytes sarcomeric organization. **d** Number of beats/30 sec observed in uninfected or SARS-CoV-2-infected cell cultures (also see supplementary movie 1). **e** IFNs and proinflammatory cytokines production in the supernatants of (**a**) at MOI 1, day 4 post-infection. **f** Replication kinetics of SARS-CoV-2 Omicron BA.1, 2 and 5 variants in cardiomyocytes. **g** Infectious SARS-CoV-2 particles in the supernatants of cardiomyocytes infected at an MOI of 0.1 and obtained at 4 days post-infection (highlighted by an arrow in panel **f**). TCID50 was determined by infection of Vero E6 cells. **h** Number of beats/30 sec observed in uninfected or SARS-CoV-2 Omicron BA.1, BA.2 and BA.5 infected cardiomyocyte cultures (also see supplementary movie 2). All panels show results obtained from three biological replicates (*n* = 3, Mean ± SEM). Significant differences compared to BA.1 are indicated as: *p < 0.05; **p < 0.01; ***p < 0.001; ns not significant

The spontaneous beating of cardiomyocytes in culture provides a sensitive indicator of cell functionality. Recording of beating behavior of cardiomyocytes, revealed rates that steadily decreased from about 30 to 10 beats per 30 s over the 10-day incubation period in uninfected cultures (Fig. 1d; Supplementary Movie 1). Cultures infected with NL-02-2020 or Delta generally stopped beating completely by day 3–5. Despite strong CPE the cultures infected with NL-02-2020 and the lowest MOI of Delta restarted some albeit locally clustered beating on days 8–10. In comparison, the effects of BA.1 on the beating activity of cardiomyocyte cultures were typically attenuated and delayed (Fig. 1d, Supplementary Fig. 5).

Induction of inflammatory cytokines plays a key role in the pathogenesis of COVID-19. Analysis of the cardiomyocyte culture supernatant obtained at 4 days post-infection revealed that NL-02-2020 and Delta induced higher levels of interferons and proinflammatory cytokines than BA.1 (Fig. 1e and Supplementary Fig. 6). For example, striking differences were detected in the induction of IL-6 and IP-10, representing important markers of disease severity and predictors of mortality in COVID-19. Despite lower levels of replication, NL-02-2020 induced higher levels of IFN-α2, IFN-γ, and IL-8 than Delta, possibly indicating that the latter evolved an increased ability to avoid innate immune activation. Altogether, these results suggest that both attenuated replication as well as lower proinflammatory cytokine induction in cardiomyocytes contribute to the reduced pathogenicity of BA.1.

Infection of human coronary artery endothelial cells (HCAEC) may contribute to COVID-19-associated cardiac disease.^8^ Thus, we also analyzed the susceptibility of this cell type to SARS-CoV-2 infection. We found that supernatants of primary HCAEC contained high levels of viral RNA 6 days after infection with Delta and BA.1, while no viral RNA was detected in cultures infected with the NL-02-2020 strain (Supplementary Fig. 7). However, in agreement with published data,^8^ the cell culture supernatant did not contain infectious virus. Irrespective of the viral dose, Delta showed higher levels of viral RNA production and stronger CPE, as well as slightly higher proinflammatory effects compared to BA.1 (Supplementary Fig. 7).

At the beginning of this study, BA.1 dominated the COVID-19 pandemic. Since then, several subvariants of Omicron emerged and outcompeted the original BA.1 VOC. BA.2 differs by a total of ~40 mutations from BA.1 and is the precursor of BA.5, which contains a deletion of H69/V70 and additional changes of L452R, F486V and R493Q in Spike and currently (August 2022) dominates the pandemic. Recent evidence suggests that BA.5 is not only more resistant to neutralizing antibodies but may also be more virulent than BA.1. We found that BA.5 replicates with faster kinetics and higher efficiency (Fig. 1f, Supplementary Fig. 8), produces more infectious virus (Fig. 1g), causes stronger CPE (Supplementary Fig. S9), and more rapidly disrupts beating (Fig. 1h, Supplementary movie 2) in cardiomyocyte cultures compared to BA.1, while BA.2 displayed an intermediate phenotype.

In summary, replication and cytopathic effects of the initial BA.1 Omicron VOC in spontaneously beating cultures of human cardiomyocytes are strongly attenuated compared to the early NL-02-2020 strain and the Delta VOC. However, BA.2 and especially BA.5 showed higher replication and caused stronger CPE than BA.1, consequently displaying features more similar to the Delta VOC. This does not come as a surprise since BA.5 shares some mutations in Spike thought to increase fusogenicity, such as L452R, with Delta. Our results add to the evidence that efficient evasion of adaptive immune responses by BA.1 came at the cost of reduced fusogenicity. However, acquisition of additional changes by BA.5 restored the full replicative potential and may potentially increase both transmissibility and virulence. Our finding that BA.1 is strongly attenuated in iPSC-derived human cardiomyocytes suggests that this variant is less likely to cause cardiac injury and cardiomyopathies compared to other SARS-CoV-2 VOCs. It will be interesting to see whether this is confirmed by patient data and if Omicron-adapted vaccines may drive the evolution of attenuated forms of BA.5 and future SARS-CoV-2 variants.

## Supplementary information

Supplementary Materials

Video 1

Video 2

## Data Availability

All data are available from the corresponding author.
