# Supplementary material for: Strong attenuation of SARS-CoV-2 Omicron BA.1 and increased replication of the BA.5 subvariant in human cardiomyocytes
Source: Signal Transduct Target Ther. 2022 Dec 25;7:395. doi: 10.1038/s41392-022-01256-9 (PMC9789943; doi:10.1038/s41392-022-01256-9)
Supplement: Supplementary file 1 — Supplementary Materials [file 41392_2022_1256_MOESM1_ESM.docx]

Supplementary Materials for

Strong attenuation of SARS-CoV-2 Omicron BA.1 and increased replication
of the BA.5 subvariant in human cardiomyocytes

Rayhane Nchioua^1^, Federica Diofano^2^, Sabrina Noettger^1^, Pascal von Maltitz^1^, Fabian Zech^1^, Steffen Stenger^2^, Jan Münch^1^, Konstantin M.J. Sparrer^1^, Steffen Just^2^ and Frank Kirchhoff^1*^

^1^ Institute of Molecular Virology, Ulm University Medical Center, Ulm, Germany

^2^ Molecular Cardiology, Department of Internal Medicine II, Ulm University, Ulm, Germany

^3^ Institute of Med. Microbiology and Hygiene, Ulm University Medical Center, Ulm, Germany

Correspondence to: [frank.kirchhoff@uni-ulm.de](mailto:frank.kirchhoff@uni-ulm.de)

**This PDF file includes:**

Materials and Methods

Supplementary figures S1 to S9

Captions for Movies S1 and S2

Supplementary References

**Other Supplementary Materials for this manuscript include the following:**

Movies S1 and S2

Materials and Methods

Cell culture

Calu-3 (human epithelial lung adenocarcinoma) cells were used to propagate the SARS-CoV-2 B.1.1.529 (Omicron) variant. Cells were cultured in Minimum Essential Medium Eagle (MEM, Sigma, Cat#M4655) supplemented with 10% (upon and after viral infection) or 20% (during all other times) heat-inactivated fetal bovine serum (FBS, Gibco, Cat#10270106), 100 units/ml penicillin, 100 µg/ml streptomycin (ThermoFisher, Cat#15140122), 1 mM sodium pyruvate (Pan Biotech, Cat#P04-8010), and 1x non-essential amino acids (Sigma, Cat#M7145). Vero E6 cells (Cercopithecus aethiops derived epithelial kidney, ATCC) were used to propagate SARS-CoV-2 NL-02-2020 and B.1.617.2 (Delta) variants and for TCID_50_ analysis. Vero E6 cells were grown in Dulbecco’s modified Eagle’s medium (DMEM, Gibco, Cat#41965039) supplemented with 2.5% (upon and after viral infection) or 10% (during all other times) heat-inactivated FBS (Gibco, Cat#10270106), 100 units/ml penicillin, 100 µg/ml streptomycin (ThermoFisher, Cat#15140122), 2 mM L-glutamine (Gibco, Cat#25030081), 1 mM sodium pyruvate (Pan Biotech, Cat# P04-8010), and 1x non-essential amino acids (Sigma, Cat#M7145). Caco-2 cells (human epithelial colorectal adenocarcinoma, kindly provided by Prof. Holger Barth (Ulm University)) were used for TCID50 analysis. Caco-2 cells were grown in the same media as Vero E6 cells but with supplementation of 10% heat-inactivated FBS. Human coronary artery endothelial cells (HCAEC; CC-2585) were purchased from Lonza and cultured in EBM-2 supplemented with 10% FCS, hydrocortisone, ascorbic acid, EGF, FGF, VEGF, R3-IGF, ascorbic acid, EGF and GA-1000 (EGM-2-Bullet kit; CC-3202, Lonza) at humidified atmosphere, at 37 °C/5% CO_2_.

SARS-CoV-2 stocks

The SARS-CoV-2 variants, B.1.617.2 (Delta); and B.1.1.529, BA.5 (Omicron BA.5), were kindly provided by Prof. Dr. Florian Schmidt and Dr. Bianca Schulte (University of Bonn). The BetaCoV/Netherlands/01/NL/2020 (NL-02-2020) lineage and hCoV-19/Netherlands/NH-EMC-1720/2021, lineage B.1.1.529 (Omicron BA.1), were obtained from the European Virus Archive. The SARS-COV-2 hCoV-19/USA/CO-CDPHE-2102544747/2021, lineage B.1.1.529, BA.2 (Omicron BA.2) was obtained from the BEI database (Cat, #NR-56520). SARS-CoV-2 strains were propagated on Vero E6 (NL-02-2020, Delta), or Calu-3 (all Omicron variants) cells. To this end, 70-90% confluent cells in 75 cm² cell culture flasks were inoculated with the SARS-CoV-2 isolate (multiplicity of infection (MOI) of 0.03-0.1) in 3.5 ml serum-free medium. The cells were incubated for 2h at 37°C, before adding 20 ml medium containing 15 mM HEPES (Carl Roth, Cat#6763.1). Virus stocks were harvested as soon as strong cytopathic effect (CPE) became apparent. The virus stocks were centrifuged for 5 min at 1,000 g to remove cellular debris, aliquoted, and stored at -80°C until further use.

Plaque-forming Unit Assay.

The plaque-forming unit (PFU) assay was performed as previously described^1^. SARS-CoV-2 stocks were serially diluted and confluent monolayers of Vero E6 cells were infected. After incubation for 2 h at 37°C with shaking every 20 min, cells were overlaid with 1.5 ml of 0.8 % Avicel RC-581 (FMC) in medium, incubated for 3 days and fixed with 4 % PFA at RT for 45 min. Subsequently, the cells were washed with PBS, incubated with 0.5 ml of staining solution (0.5 % crystal violet and 0.1 % triton in water) for 20 min at RT. Finally, the staining solution was removed using water, virus-induced plaque formation quantified, and PFU per ml calculated.

Tissue Culture Infection Dose50 (TCID_50_) endpoint titration

SARS-CoV-2 stocks or infectious supernatants were serially diluted on Caco-2 (15,000) or Vero E6 (8,000) cells. Caco-2 or Vero E6 cells were seeded per well in 96 F-bottom plates in 100 µl medium and incubated overnight. Next, 100 µl of diluted SARS-CoV-2 stocks or infectious supernatants were used for infection, resulting in final dilutions of 1:10^1 to 1:10^12 on the cells in 8 (for Caco-2 cells) or 6 technical replicates (for Vero E6 cells). Cells were incubated for 7 days and monitored for CPE. TCID50/ml was calculated according to the Reed and Muench method.

Cardiomyocyte differentiation

Human episomal hiPSCs (#A18945, Thermo Fisher Scientific) at passage 2 were split using TrypLE (#12604-013, Thermo Fisher Scientific) to generate a single cell suspension. 150.000 iPS cells were seeded on Geltrex (#A1413302, Thermo Fisher Scientific) matrix coated 12 well plates. When the cells reached 80-90% confluency, differentiation protocol into ventricular cardiomyocytes using the Kleinsorge *et al*. protocol^2^ was initiated. Subsequently, to further increase the purity of the cardiomyocyte culture, metabolic selection using glucose-depleted culture medium supplemented with lactate was applied^2,3^. After metabolic selection, successful differentiation into cardiomyocytes is routinely verified by FACS analyses as reported by Luecke *et al*.^4^ with anti-SIRPA–IgG-phycoerythrin-Cy7 (clone SE5A5; BioLegend). This method yields a homogeneous population of ventricular cardiomyocytes from human iPSCs with a purity of ~90%. Cardiomyocytes start to contract 12 days after the initiation of differentiation.

Cardiomyocytes infection and beating kinetics

Human iPSC-derived ventricular cardiomyocytes were cultured in 12 wells plates. Cells were infected with increasing MOIs (0.01, 0.1 and 1) of SARS-CoV-2 variants. At 5 h post-infection, input virus was removed and cells were supplemented with fresh media, and Day 0 control was collected as background control. Virus-containing supernatant was harvested every day, or every two days (as indicated) and replaced with fresh media until day 10 post-infection. N gene RNA copies were determined by qRT-PCR for each harvested time point. The beating activity of cardiomyocytes was determined by calculating beat-to-beat activity caused by their contraction. Representative videos were recorded by Leica DM IL LED microscope using LAS X life software) in intervals of 30 seconds (at ~10 frames per second), every 24h during infection kinetics. The number of beats was determined blindly by two independent examiners.

qRT-PCR

N (nucleoprotein) RNA levels were determined in supernatants collected from SARS-CoV-2 infected cultures. Total RNA was isolated using the Viral RNA Mini Kit (Qiagen) according to the manufacturer’s instructions. qRT-PCR was performed according to the manufacturer’s instructions using TaqMan Fast Virus 1-Step Master Mix (Thermo Fisher) and a OneStepPlus Real-Time PCR System (96-well format, fast mode). Primers were purchased from Biomers and dissolved in RNAse-free water. Synthetic SARS-CoV-2-RNA (Twist Bioscience) was used as a quantitative standard to obtain viral copy numbers. All reactions were run in duplicates. (Forward primer (HKU-NF): 5’-TAA TCA GAC AAG GAA CTG ATT A-3’; Reverse primer (HKU-NR): 5’-CGA AGG TGT GAC TTC CAT G-3’; Probe (HKU-NP): 5’-FAM-GCA AAT TGT GCA ATT TGC GG-3’TAMRA).

iPSC derived-cardiomyocytes immunostaining and confocal imaging

Fully differentiated cardiomyocytes were plated onto 96-well glass plates at a cell density of 5x10^4^ cells/well. At 3 days post-infection, the cells were fixed with 4% PFA in PBS for 30 min at room temperature. After fixation, 1% saponin in PBS was added to each well (15 min at room temperature) to permeabilize the cells. The solution was removed, and the Blocking Solution (3% BSA in PBS) was added to each well, followed by an incubation of 30 min. A specific volume of the primary antibody was added directly to the Blocking Solution covering the cells and incubated overnight at 4 °C. The morning after, the cells were washed 2-3 times using PBS. The appropriate secondary antibody was added, diluted in the Blocking Solution, and incubated for 1 hour at room temperature, followed by other 3 washing steps with PBS. During the last washing step, 2 drops of NucBlue® Fixed Cell Stain (Thermo Fisher #R37606) were added to each well and incubated for 5 min. After, the cells were newly washed and stored in PBS. The antibody used were: anti-Cardiac Troponin T (1:400, Thermo #A25969), anti-alpha-Actinin (1:200, Sigma #A7811), Monoclonal Anti-SARS-CoV S Protein (1:200, Genetex # GTX635692), Alexa Fluor 488 anti-mouse IgG1 (1:1000, Invitrogen #A21121), Alexa Fluor 555 anti-rabbit IgG (1:1000, Invitrogen #A21429). Images of the stained cells were acquired using the Leica TCS SPE-II DMi8 confocal microscope.

Multiplex ELISA

Cytokine ELISA (LEGENDplex™ Human Anti-Virus Response Panel; BioLegend #740390) was performed according to the manufacturer´s instructions. In short, 20 µl of supernatants from uninfected (mock) and SARS-CoV-2 infected cells were incubated with beads coated with primary antibodies against the indicated cytokines. After incubation with secondary, fluorescently labeled antibodies, the beads were harvested and fixed in 1% PFA for 30 min at room temperature. The fixed beads were analyzed on a BD Canto II flow cytometer. Cytokines levels were quantified in the LEGENDplex Software v.8.0 using the provided standard.

HCAEC infection and kinetics

HCAEC were cultured in 12 wells plates. Cells were infected with increasing MOIs (0.01, 0.1 and 1) of the different SARS-CoV-2 variants. At 5 h post-infection, cells were washed once with PBS to remove input virus and supplemented with 1ml fresh media, and Day 0 control was collected as wash control. Supernatants were harvested every 2 days and replaced with fresh media until day 6 post-infection. N gene RNA copies were determined by qRT-PCR for each harvested time point, as well as TCID50 analysis for day 6 post-infection of harvested supernatants.

Statistical analysis

Statistical analysis was performed using GraphPad Prism software. Unpaired students t-test was used for all statistical analysis performed, except for TCID_50_, where ratio paired students t-test was used. Significant differences are indicated as: *, p < 0.05; **, p < 0.01; ***, p < 0.001.


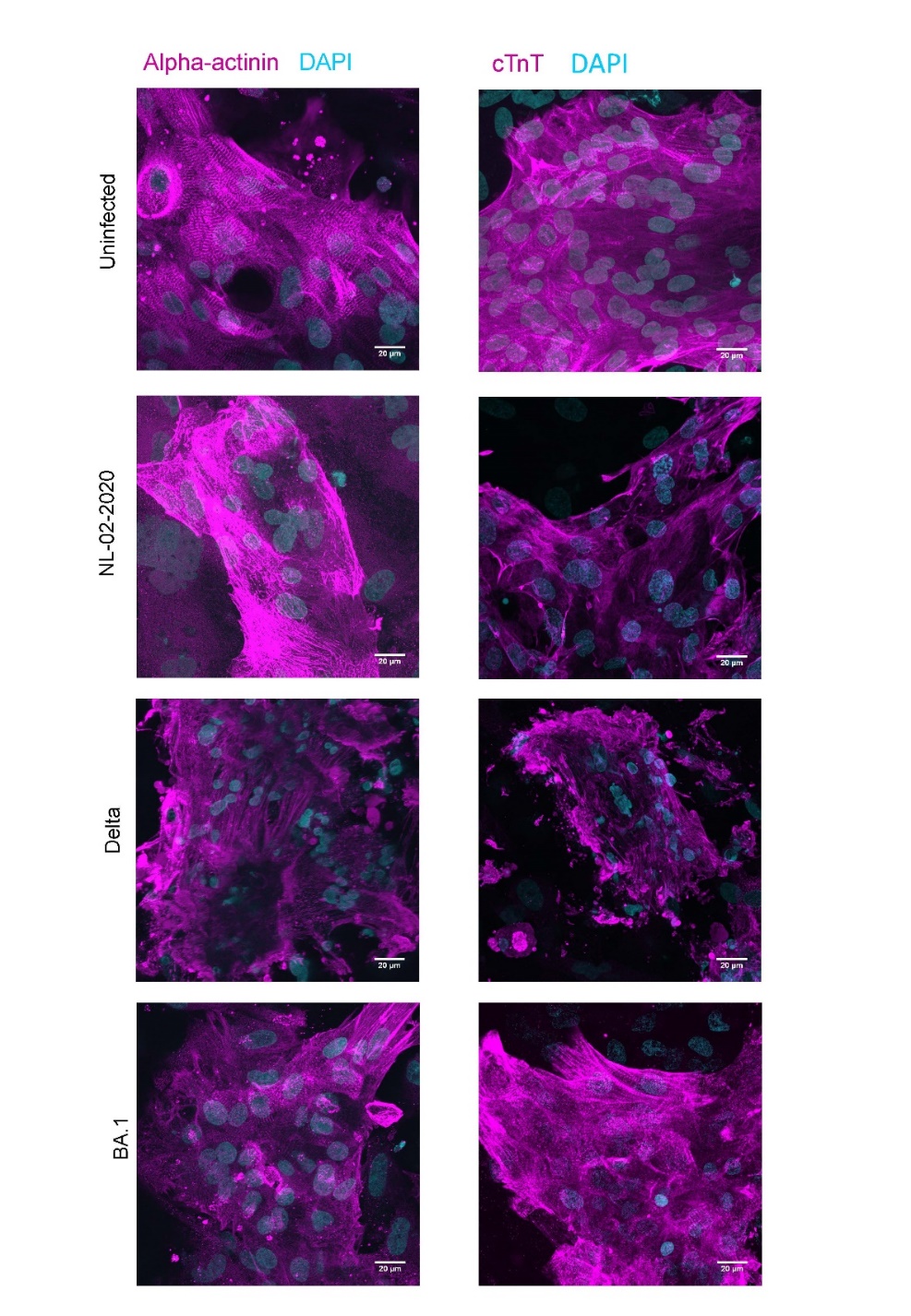


**Figure S1. Expression of cardiac Troponin T and alpha-actinin by cardiomyocyte cultures.**

Uninfected iPSC-derived cardiomyocytes and cells infected with the indicated SARS-CoV-2 variants (MOI of 1) were co-immunostained at day 3 post-infection for alpha-actinin (magenta) or cardiac Troponin T (cTnT, magenta) and DAPI (cyan). Stainings show the efficient differentiation of human iPSCs into ventricular cardiomyocytes. Scale bar 20 µm.


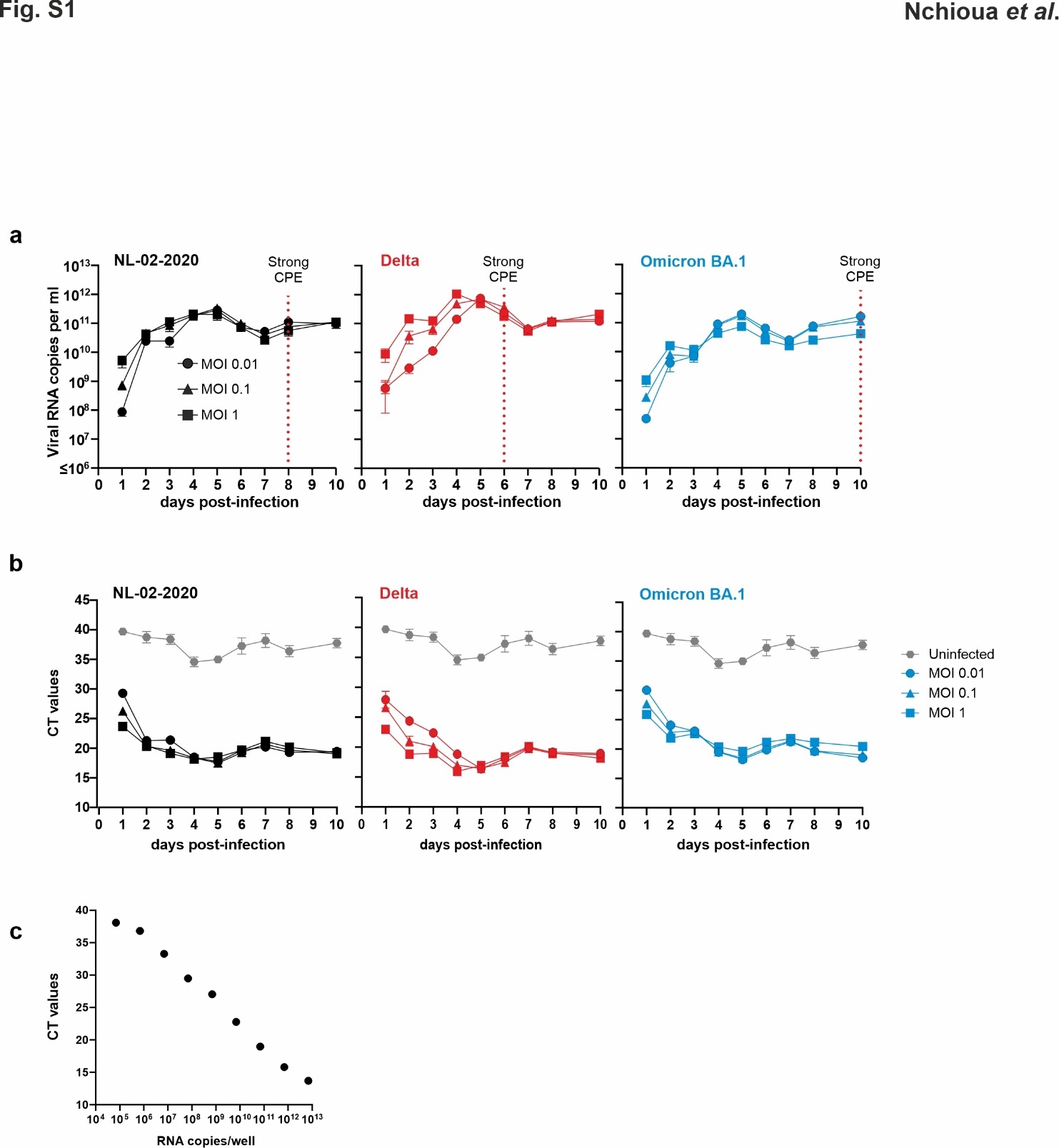


Figure S2. Replication kinetics of indicated SARS-CoV-2 variants in human cardiomyocytes.

**a,** Quantification of viral N RNA levels in the supernatant of SARS-CoV-2 infected cardiomyocytes with increasing MOIs (as indicated), at indicated days post-infection. Shown are results of three biological replicates (n=3, Mean±SEM). **b,** Raw qRT-PCR CT values corresponding to (a) (n=3, Mean±SEM). **c,** Standard curve.

**
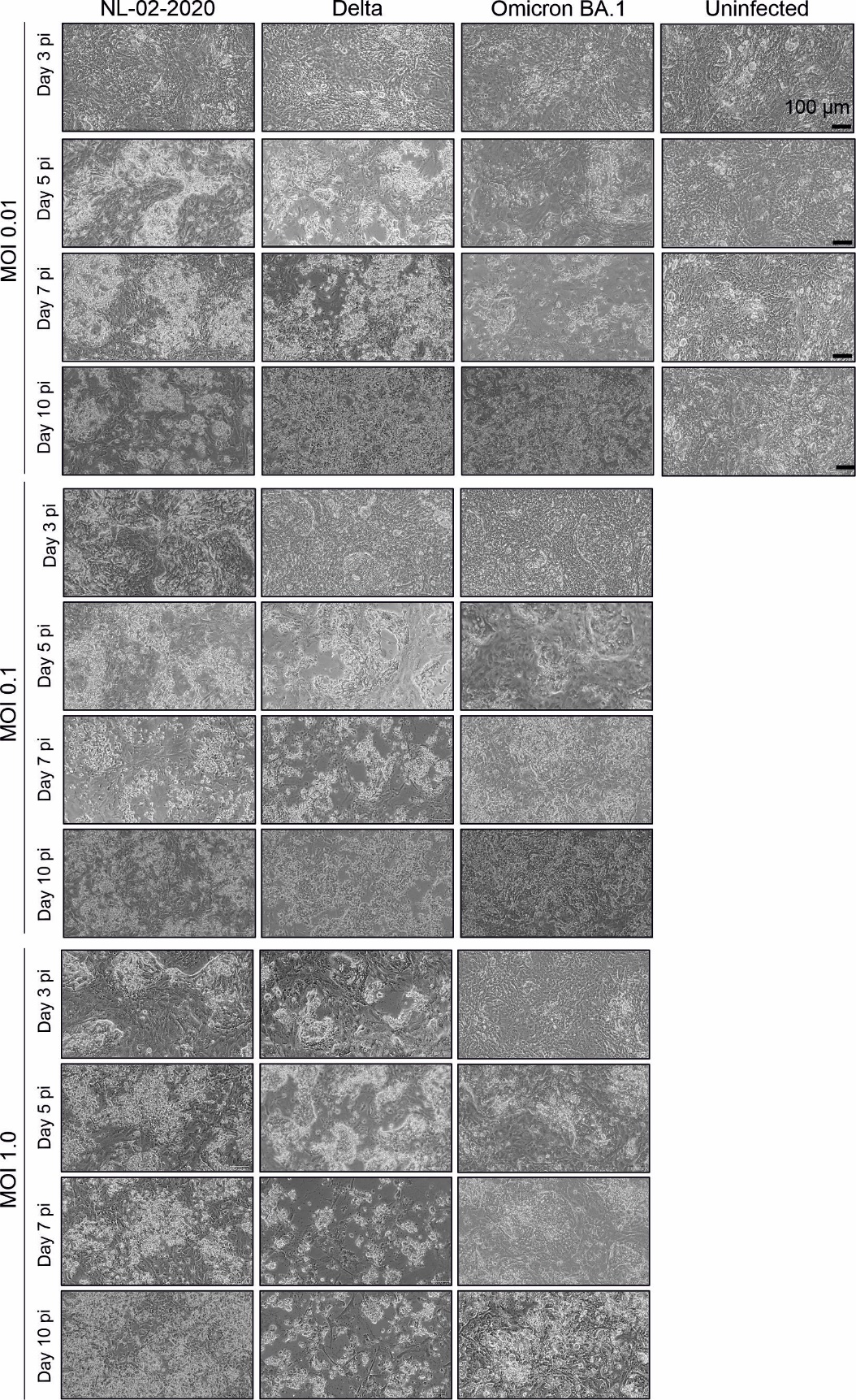
**

Figure S3. Cytopathic effects in SARS-CoV-2-infected cardiomyocyte cultures.

Representative images of cytopathic effects in cardiomyocyte cultures infected with different SARS-CoV-2 variants at the indicated MOIs. Images were taken at indicated days post-infection (pi) and the corresponding uninfected control cultures. Scale bar 100 µm.


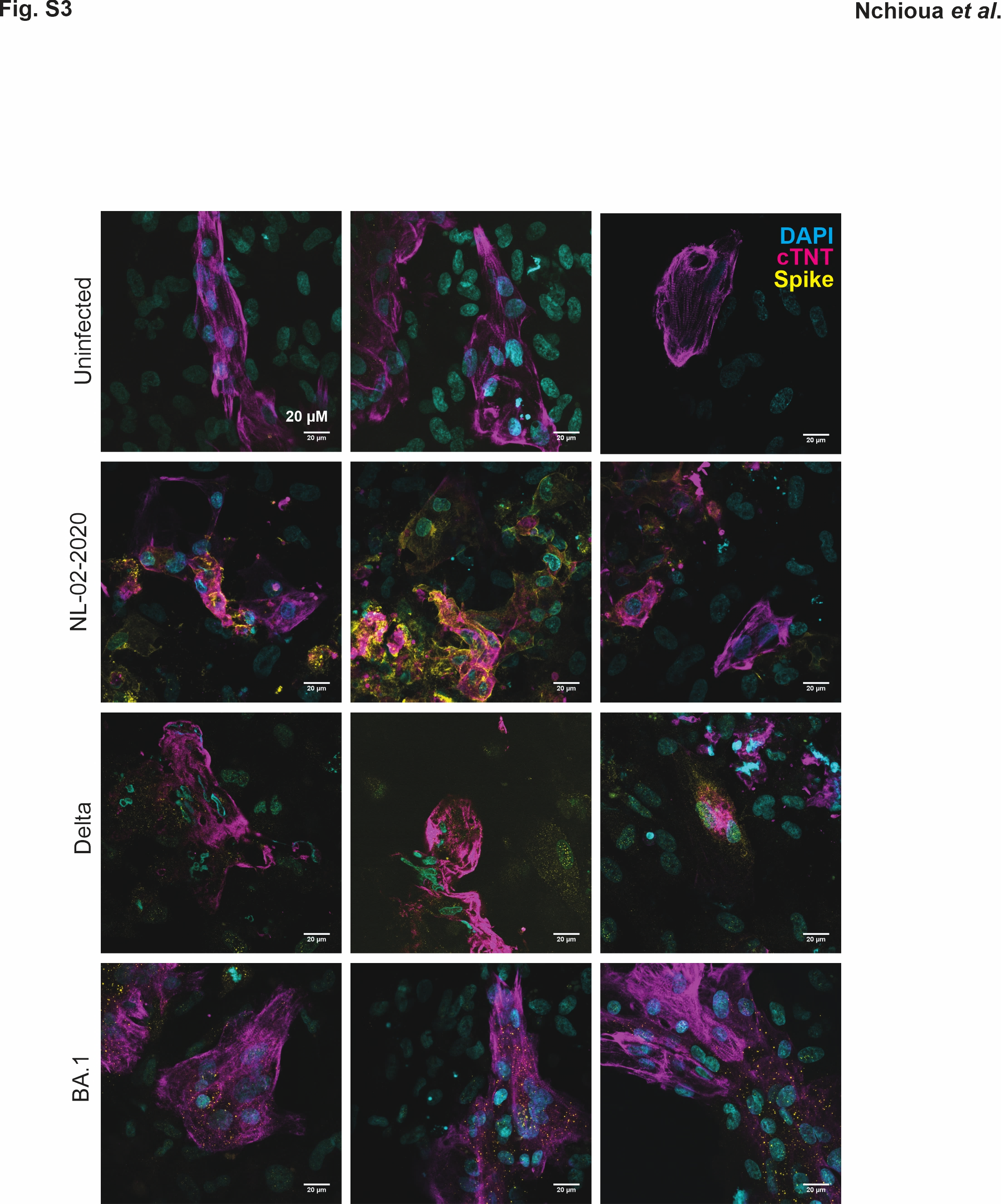
Figure S4. Effects of SARS-CoV-2 infection on iPSC- derived cardiomyocytes.

Uninfected cardiomyocytes or cells infected with the indicated SARS-CoV-2 variants (MOI of 1) for 3 days were stained for cardiac Troponin T (cTnT, magenta), the SARS-CoV-2 Spike protein (yellow), and the nucleus (DAPI, cyan). Scale bar 20 µm.


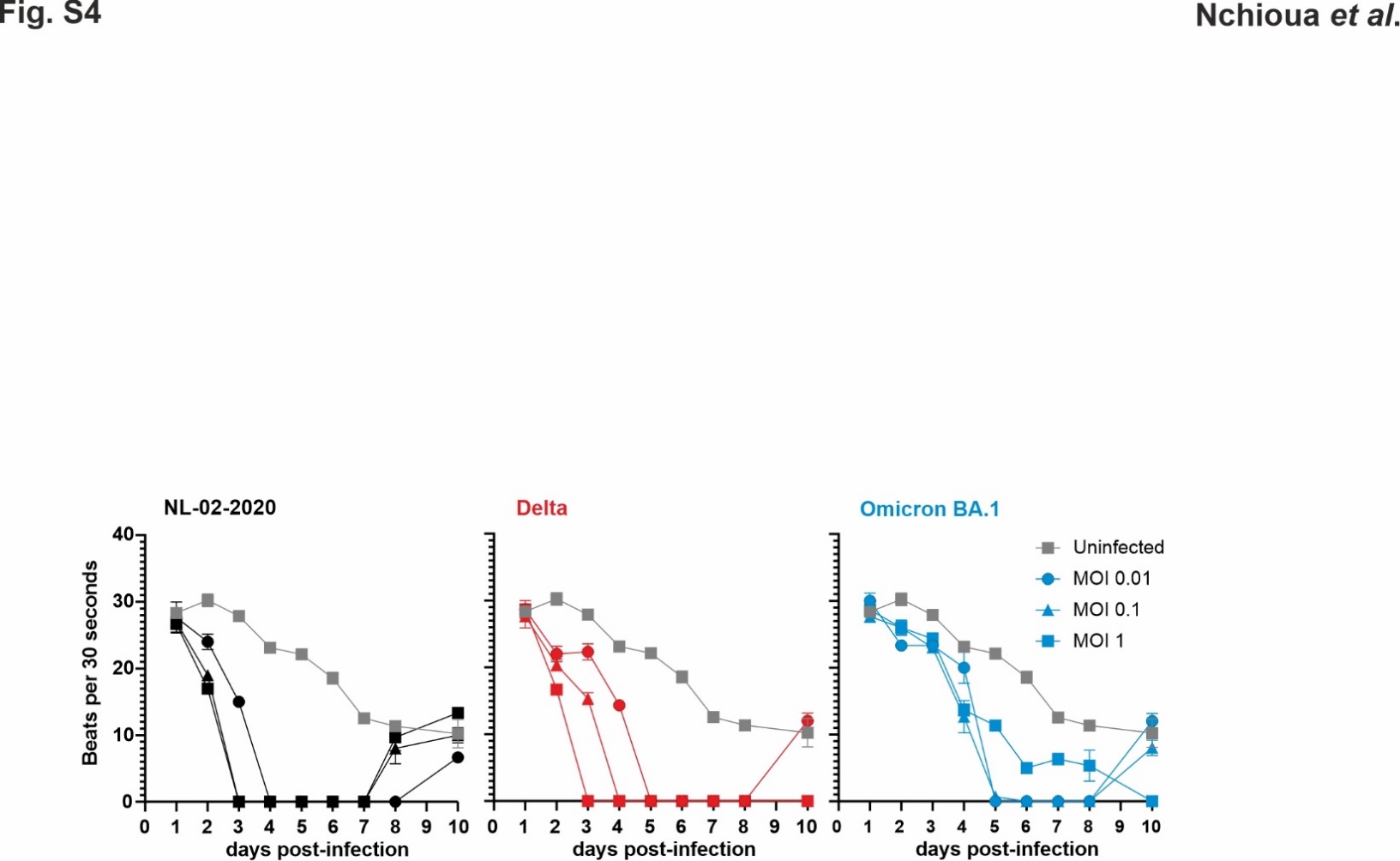
Figure S5. Impact of infectious dose of SARS-CoV-2 variants on the beating activity of cardiomyocytes.

Number of beats/30 seconds observed in the respective uninfected or SARS-CoV-2-infected cell cultures at indicated days post-infection. Data represent mean numbers of beats of three biological replicates (n=3, Mean±SEM). Data correspond to those shown in Fig. 1d, highlighting differences between the different SARS-CoV-2 variants.


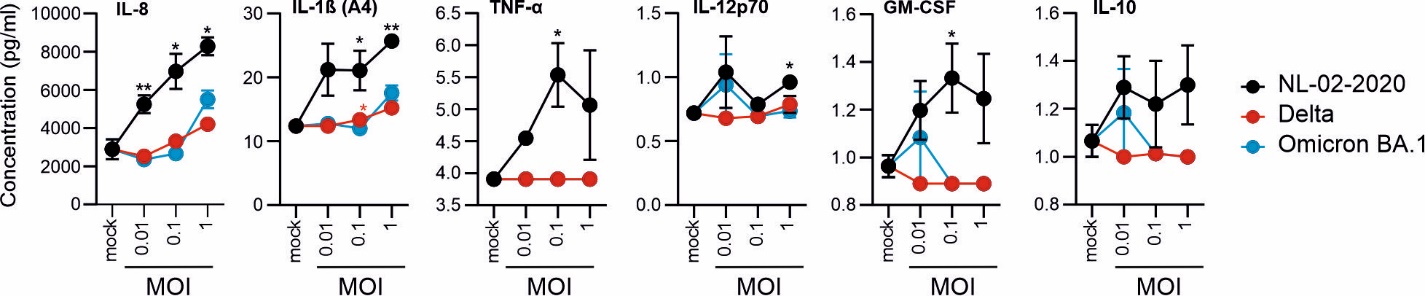
Figure S6. Cytokines production by infected cardiomyocytes.

Pro-inflammatory cytokines in the supernatants of uninfected control cells and cardiomyocytes infected with the indicated SARS-CoV-2 variants obtained at day 4 post-infection. (n=3, Mean±SEM). Significant differences compared to BA.1 are indicated: *, p < 0.05; **, p < 0.01.


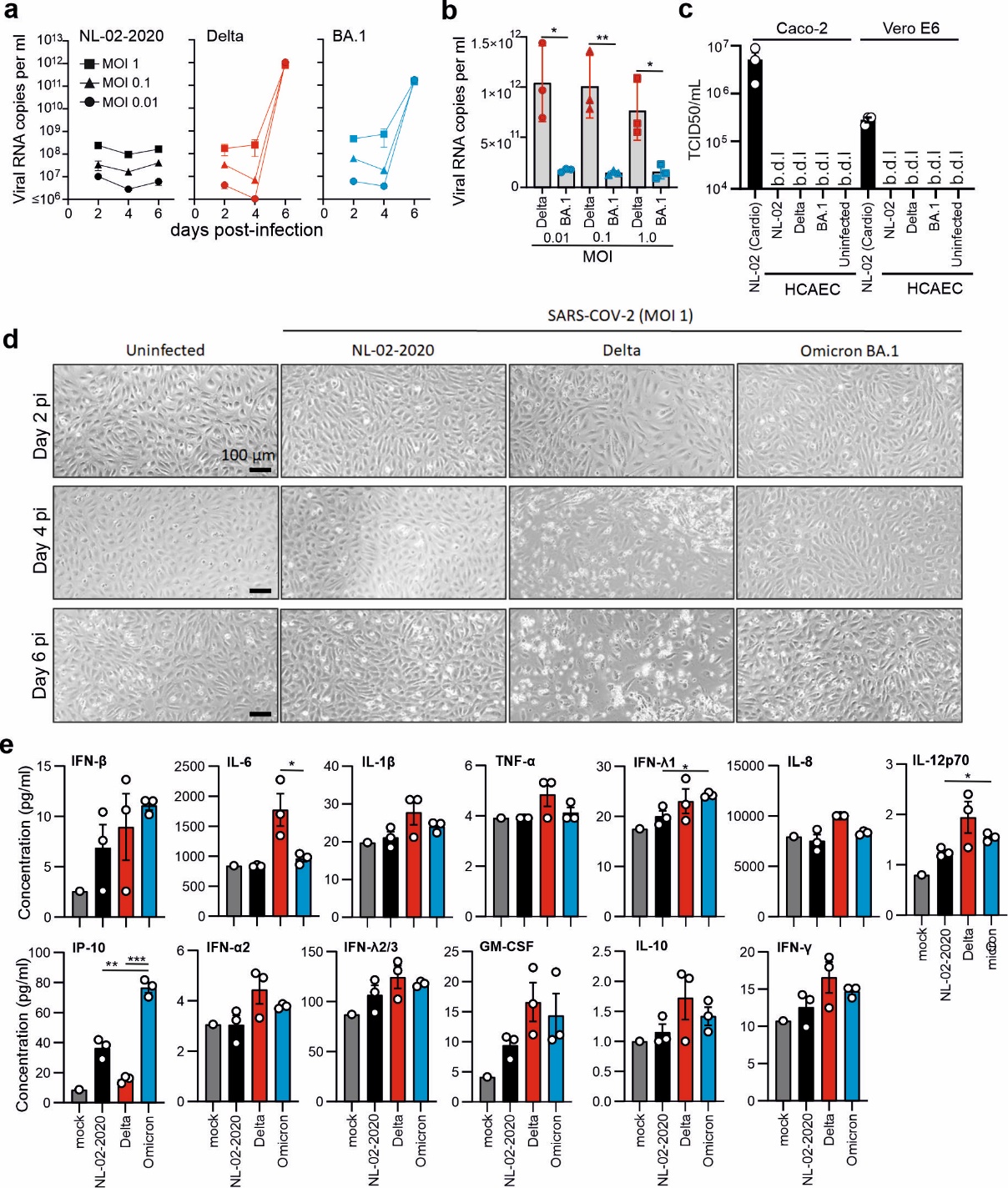


Figure S7. Infection of human coronary artery endothelial cells by SARS-CoV-2 variants.

**a,** Viral RNA production by HCAEC cells at the indicated days post-infection with SARS-CoV-2 NL-02-2020, Delta or BA.1. **b,** Comparison of viral RNA levels in the supernatant of HCAEC cultures infected with the Delta or BA.1 VOCs at 6 days post-infection as shown in panel a. **c,** Infectious SARS-CoV-2 particles in the supernatants of HCAEC cells infected at an MOI of 1 and obtained at 6 days post-infection. TCID50 was determined by infection of Caco-2 and Vero E6 cells. Supernatants from cardiomyocytes infected with NL-02-2020 at 4 or 6 dpi (see Fig. 1b) are shown as positive control. b.d.l, below detection limit. **d,** Cytopathic effects of SARS-CoV-2 on infected HCAEC cultures. **e,** IFNs and pro-inflammatory cytokines in the supernatants of HCAEC cells infected with the indicated SARS-CoV-2 variants (MOI of 1) at 6 days post-infection. Significant differences are indicated: *, p < 0.05; **, p < 0.01; ***, p < 0.001 (n=3, Mean±SEM).


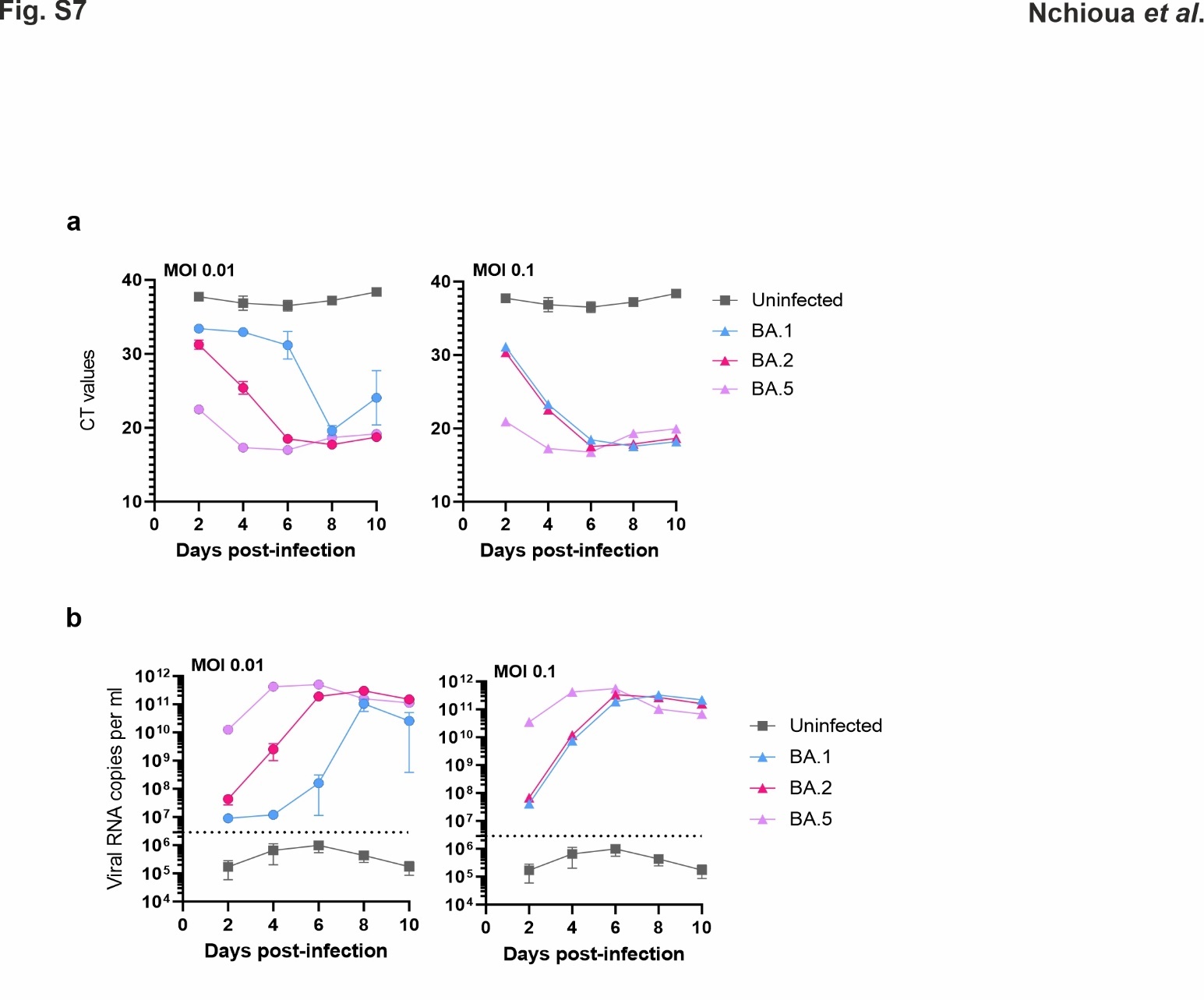


Figure S8. Replication of SARS-CoV-2 Omicron variants in human cardiomyocytes.

**a,** Raw qRT-PCR CT values and **b**, quantification of viral N RNA levels in the supernatants of SARS-CoV-2 infected cardiomyocytes at the indicated days post-infection. Shown are results of three biological replicates (n=3, Mean±SEM). The dotted line indicates the detection limit of the assay.


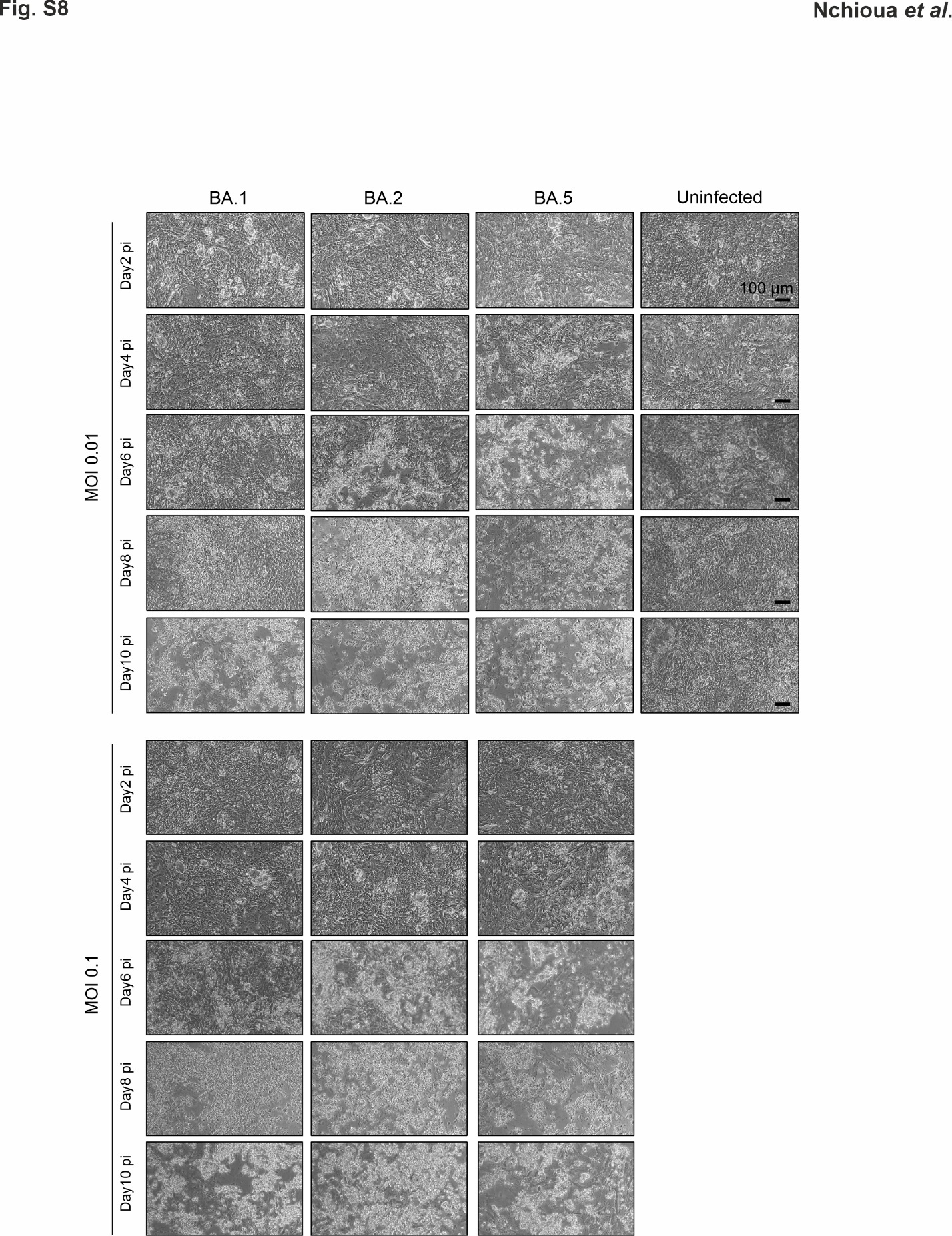


Figure S9. Cytopathic effects in SARS-CoV-2 Omicron infected cardiomyocyte cultures.

Representative images of cytopathic effect of infected cardiomyocytes, with indicated MOIs, of different SARS-CoV-2 Omicron variants. Images were taken at indicated days post-infection, as well as their respective uninfected controls. Scale bar 100 µm.

Captions for movies

Movie S1. Impact of SARS-CoV-2 NL-02-2020, Delta and BA.1 infection on cardiomyocytes.

Representative videos were recorded by a Leica DM IL LED microscope using LAS X life software in intervals of 25 seconds (at ~10 frames per second), every 24 hours during the replication analysis shown in Fig. 1a. The frequency of beats in the cultures shown in supplementary movie 1 is provided in Fig. 1d. Supplementary Movie 1 shows cardiomyocytes that were left uninfected or infected with SARS-CoV-2 variants NL-02-2020, Delta and BA.1 (MOI of 1). Viral strains and times post-infection are indicated in the upper left corner of the movies. The cultures correspond to the experiment shown in Fig. 1a-e. Twelve clips of 25s each were combined using Adobe Premiere Pro 2022. The caption in the top left corner states viral strain and day after infection. The files were exported at 10 frames per second, in 720 x 480 format to reduce size.

Movie S2. Impact of SARS-CoV-2 BA.1, BA.2 and BA.5 infection on cardiomyocytes.

Representative videos were recorded by a Leica DM IL LED microscope using LAS X life software in intervals of 25 seconds (at ~10 frames per second), every 48 hrs during the replication analysis shown in Fig. 1f. The frequency of beats in the cultures shown in supplementary movie 2 is provided in Fig. 1h. Supplementary movie 2 shows cardiomyocytes that were left uninfected or infected with SARS-CoV-2 BA.1, BA.2 or BA.5 at an MOI of 0.01 for the time periods indicated in the upper left corner of the movies. Eight clips of 25s each were combined using Adobe Premiere Pro 2022. The caption in the top left corner states the viral strain and day after infection. The files were exported at 10 frames per second, in 720 x 480 format to reduce size.

Supplementary References

1. Nchioua, R. *et al.* SARS-CoV-2 Is Restricted by Zinc Finger Antiviral Protein despite Preadaptation to the Low-CpG Environment in Humans. *mBio* **11**, 16 (2020).

2. Kleinsorge, M. & Cyganek, L. Subtype-Directed Differentiation of Human iPSCs into Atrial and Ventricular Cardiomyocytes. *STAR Protoc* **1**, 100026 (2020).

3. Cyganek, L. *et al.* Deep phenotyping of human induced pluripotent stem cell-derived atrial and ventricular cardiomyocytes. *JCI Insight* **3**, 99941 (2018).

4. Luecke L. *et al*. Reliable Protocols for Flow Cytometry Analysis of Intracellular Proteins in Pluripotent Stem Cell Derivatives: A Fit-For-Purpose Approach. *Curr. Protoc. Stem Cell Biol*. **50**, e94 (2019).
